# Western Medical Society of the State of Wisconsin

**Published:** 1850-05

**Authors:** Geo. D. Wilber

**Affiliations:** Recording Secretary


### ARTICLE VII.

### *Western Medical Society of the State of Wisconsin.*

A Convention of Physicians and Surgeons from the counties of Iowa, Grant, and La Fayette, and State of Wisconsin, assembled in Shullsburg, La Fayette Co., the 4th day of December, 1849, and proceeded to organize a Medical Society, which is called the "Western Medical Society of the State of Wisconsin," and the following named persons were elected for one year, to the various offices respectively, as follows:

Dr. J. W. Clark, of Plattville, President; Dr. A. P. Ladd, of Shullsburg, Vice President; Dr. Geo. D. Wilber of Mineral Point, Recording Secretary; Dr. J. S. Russell of Plattville, Corresponding Secretary; Dr. G. W. Phillips of Dodgeville, Treasurer; Dr. H. Van Dusen of Mineral Point, Dr. A. P. Ladd, of Shullsburg, and Dr. J. W. Clark of Plattville, Censors.

A series of By-laws, Orders, &c., were adopted by the Society, and also the Code of Medical Ethics, recommended by the National Medical Convention in 1847. Several of the popular movements of the day, were touched, without any definite action upon them.

Resolutions approving of the National Medical Conventions, and American Medical Associations, were introduced and unanimously adopted, and a copy of their proceedings ordered for the Society. Dr. H. Van Dusen, was chosen delegate to attend the latter at its next meeting.

The best feeling pervaded the meeting, and all se emed determined to stop the inroads of quackery in any of its various forms; to assist in promoting the harmony and interests of the Profession of Medicine, and in elevating the standard of Medical Education.

The Society holds its sessions semi-annually, and its next meeting will be held at Mineral Point, the first Tuesday of June, 1850.

GEO. D. WILBER, M.D., *Recording Secretary.*

